# The Hurdles From Bench to Bedside in the Realization and Implementation of a Universal Influenza Vaccine

**DOI:** 10.3389/fimmu.2018.01479

**Published:** 2018-07-02

**Authors:** Sophie A. Valkenburg, Nancy H. L. Leung, Maireid B. Bull, Li-meng Yan, Athena P. Y. Li, Leo L. M. Poon, Benjamin J. Cowling

**Affiliations:** ^1^HKU Pasteur Research Pole, The University of Hong Kong, Pokfulam, Hong Kong; ^2^WHO Collaborating Centre for Infectious Disease Epidemiology and Control, School of Public Health, The University of Hong Kong, Pokfulam, Hong Kong

**Keywords:** influenza virus, universal vaccine, T cell, hemagglutinin-stalk, clinical trials

## Abstract

Influenza viruses circulate worldwide causing annual epidemics that have a substantial impact on public health. This is despite vaccines being in use for over 70 years and currently being administered to around 500 million people each year. Improvements in vaccine design are needed to increase the strength, breadth, and duration of immunity against diverse strains that circulate during regular epidemics, occasional pandemics, and from animal reservoirs. Universal vaccine strategies that target more conserved regions of the virus, such as the hemagglutinin (HA)-stalk, or recruit other cellular responses, such as T cells and NK cells, have the potential to provide broader immunity. Many pre-pandemic vaccines in clinical development do not utilize new vaccine platforms but use “tried and true” recombinant HA protein or inactivated virus strategies despite substantial leaps in fundamental research on universal vaccines. Significant hurdles exist for universal vaccine development from bench to bedside, so that promising preclinical data is not yet translating to human clinical trials. Few studies have assessed immune correlates derived from asymptomatic influenza virus infections, due to the scale of a study required to identity these cases. The realization and implementation of a universal influenza vaccine requires identification and standardization of set points of protective immune correlates, and consideration of dosage schedule to maximize vaccine uptake.

## Introduction

Influenza A viruses have over 18 different hemagglutinin (HA) subtypes, and continual antigenic drift of seasonal H3N2 and H1N1 viruses generates new variants. In addition, there are distinct lineages of influenza B viruses that also exhibit antigenic drift, meaning there is a plethora of influenza viruses that pose a threat to public health (1). Reports of global influenza infection rates estimate that up to 18% of the population can be infected during annual influenza epidemics (2), causing excess morbidity and mortality resulting in projected economic losses of nearly US$87 billion (3). Influenza vaccines are the most widely used vaccines in the world due to annual updates on circulating strains and health authority recommendations for at risk groups (4). The groups most commonly targeted for influenza vaccination programs are children and elderly, pregnant women, immunocompromised, and healthcare workers (HCWs). Inactivated influenza vaccines (IIV) administered intramuscularly have been available since the 1940s and progressive developments have been made to increase breadth of immunity provided by the vaccine, from monovalent to bivalent and then trivalent, to most recently quadrivalent formulations (5). The use of split and subunit vaccines has provided a more purified formulation, and the use of improved adjuvants with reduced side effects in recent years, such as MF59 and AS03 has enabled antigen sparing and increased immunogenicity of vaccine antigens (5, 6). One important advance was the release of live-attenuated influenza vaccines (LAIV) by MedImmune to American markets in 2003, delivered as a nasal spray (7). The quadrivalent cell-grown recombinant HA protein vaccines, FluCelVax (Seqirus) available from 2012 (8) and FluBlok (Sanofi Pasteur) available from 2013 (9), provide an expedient pipeline for pandemic vaccine responsiveness and avoid egg adaptations generated during vaccine production.

A combination of issues exists for the current influenza vaccines (10), including egg adaptations (11), lag between strain selection and vaccine availability (10), and breadth and duration of immunity (12). Annual vaccine effectiveness (VE) is variable and contingent upon antigenic distance between vaccine and circulating strains and the individual’s immune history (13). Shortcomings in VE for IIV and LAIV are repeatedly reported with a recent average VE found to be 78.4% and 30.7% against H1N1pdm09 infections, respectively, in 2- to 17-year olds in 2015/2016 in the UK, US, Canada, and Finland (14), while IIV VE reported by the CDC ranges from 10 to 60% from 2004 to 2016 (15). Therefore, current vaccines are not effective enough, with negative or low VE reported, and LAIV does not appear to improve upon VE over IIV consistently (16–18), hence, the need for universal vaccines. Furthermore, current IIV VE decreased over time by one-third from 3 to 6 months post vaccination (19), and reduced VE estimates over time were seen for LAIV (20, 21). Targeting the elderly for vaccination is a logical step as they are the demographic that have the highest morbidity and mortality risk from an influenza virus infection; however, current vaccines are even less effective at conferring protection within this susceptible age group (22).

## Criteria for Design of Next-Generation Universal Influenza Vaccines

WHO published in 2017 the Preferred Product Characteristics for Next-Generation Influenza Vaccines which lays out the targets for influenza vaccine development over the next 5 and 10 years (23). In the first 5 years, the WHO encourages the evaluation of currently available vaccine and vaccine technologies to achieve greater protection against vaccine-matched or drifted influenza strains and protection against severe influenza for at least 1 year. In 10 years, by 2027, the WHO encourages research and development in next-generation vaccines to provide universal protection against severe influenza A illness for at least 5 years. In addition, a strategic work plan for the design of a universal vaccine has been outlined by the NIH NIAID (10, 24, 25). To achieve the goal of a universal influenza vaccine capable of providing protection beyond 1 year and with broader immunity against antigenically diverse strains, the work plan identified areas for expanded research efforts to address this goal, with an emphasis on research in the areas of (1) influenza transmission, natural history, and pathogenesis; (2) development of influenza immunity and correlates of protection; and (3) rational design of universal influenza vaccines.

Ultimately, an ideal universal influenza vaccine would provide protection (1) against seasonal influenza epidemics by drift variants between seasons or pan-influenza A and B viruses, (2) against influenza pandemics with limited prior population immunity, and (3) against zoonotic (e.g., avian) influenza infections with severe disease outcomes. However, this “ideal” vaccine is still stuck at the laboratory bench (26), with fundamental questions about immune correlates of protection required for universal protection against influenza viruses yet to be answered.

## Strategies to Increase the Strength, Duration, and Breadth of Vaccine-Induced Immune Responses

### Timing of Priming for T Cell Immunity

Existing inactivated virus-based vaccine technologies could be improved to increase the strength and duration of the vaccine-induced responses to overcome seasonal influenza epidemics of drifted variants. While natural infection may generate protective immunity for 2–10 years (2, 27), it has been reported that IIV sero-protection fell below 60% one year after vaccination (28). Therefore, bridging the gap between protection afforded by infection and vaccination, by defining immune correlates of protection (Table 1) associated with better outcomes of natural infection, severity of infection, and the protection afforded by current and other next-generation vaccines in clinical development (Table 2) is critical to future vaccine design.

**Table 1:** Broadly reactive correlates of protection from symptomatic influenza virus infection from human studies.

Reference	Sample size	Age (years)	Time points	Infection/vaccination	Immune correlate	Findings
**Infection studies**						
Hayward et al. 2015 (2)	1,414, and 205 cases	0–65+	2006–2011, pre and post season	Natural infection (pdmH1N1)	CD8^+^ and CD4^+^ T cells	Prior T cell immunity correlates with reduced viral shedding
Sridhar et al. 2013 (29)	342, and 25 cases	18–64	2009–2011, recruitment, 6, 12, and 18 months	Natural infection (pdmH1N1)	CD8^+^ T cells	Prior CD8^+^ T cell immunity correlates with reduced viral shedding, 10-fold response increase results in 7-fold decrease risk of infection
Couch et al. 2013 (30)	1,509, and 226 cases	18–49	2009–2011, pre and post season	Natural infection (H1, H3, FluB)	Hemagglutination inhibition (HAI) and neuraminidase (NAI)	NAI and HAI are independent correlate of protection and NAI correlates with reduced symptoms
Johnstone et al. 2014 (31)	1,072, and 21 cases	>65	2009–2011, pre and post season	Natural infection	Treg	High Treg correlated with reduced infection, and high CMV + CD4^+^ T cells correlated with increased risk of infection
Monto et al. 1973 (32)	274	<45	Recruitment, 6, and 12 months	Natural infection (H3N2)	NAI	No detectable NAI response correlates with increased infection
Aho et al. 1976 (33)	90	20–71	Pre and post season	Natural infection (H3N2)	Secreted IgA (sIgA)	IgA deficiency and lack of HAI serum rise correlated with increased symptoms scores
Savic et al. 2017 (34)	150 pregnant women	17–42	Recruitment	Natural infection (pdmH1N1)	CD8^+^ and CD4^+^ T cells, NK cells	Lower symptoms associated with higher late effector and naïve CD8^+^ T cells, multifunctional CD4^+^ T cells and lower NK cells
Oshansky et al. 2014 (35)	84	0–18+	0, 3, 7, 10, and 28 days	Natural infection (hospitalized vs non-hospitalized)	Monocytes and cytokines	Conventional monocytes vs patrolling monocytes and elevated IL-10, MCP3, IL-6 cytokines
Agrati et al. 2010 (36)	28	3–69	Acute and day 20–27 post admission	Natural infection (severe vs mild)	CD4^+^ T cells	Lymphopenia resulted in severe infection, reduced CD4^+^ T cells in circulation, increased TCM, TEM, reduced TN and apoptotic CD95^+^
Fox et al. 2012 (37)	49	19–57	0, 2, 5, 10, 14, and 28 days post admission	Natural infection (severe vs mild)	NK cells, CD4^+^, and CD8^+^ T cells	Lymphopenia resulted in severe infection
Zhao et al. 2012 (38)	48	18–65	2–3 days post hospital admission	Natural infection (severe vs mild)	HAI, CD8^+^ and CD4^+^ T cells, monocytes, and cytokines	Severe infections had greater HAI titers and increase T cell response post-infection, reduced IL-17, increased GMCSF in severe group
Wong et al. 2018 (39)	52	12–78	Acute and day 14 post admission	Natural infection (severe vs mild)	HAI, CD8^+^ and CD4^+^ T cells, monocytes, and cytokines	Delay in T cell recruitment, prolonged activation, high pro-inflammatory cytokines and reduced regulation of T cell responses correlate with severe infection
Wang et al. 2015 (40)	16	47–88	10, 21, and 30 days	Natural infection (H7N9 survived vs fatal)	CD8^+^ and CD4^+^ T cells, NK cells	Delay in CD4^+^ and CD8^+^ T cell and NK cell recruitment in fatal cases
Vanderven et al. 2017 (41)	34	22–88	Admission and death/release	Natural infection (H7N9 vs seasonal)	ADCC	Fc effector functions (ADCC) precede nAb responses
Diao et al. 2014 (42)	23	18–65	Daily, 0–31 days post admission	Natural infection (H7N9 mild vs severe)	T cells, monocytes, cytokines	Lymphopenia resulted in severe infection and reduced T cells, monocytes and cytokines. HLA-DR^+^ on CD14^+^ negatively correlate with severity
McMichael et al. 1983 (43)	63	18–47	0, 5, and 14–21 days	Experimental infection	CD8^+^ T cells	Prior T cell immunity (by birth year) and low HAI and NAI correlates with reduced viral shedding
Wilkinson et al. 2012 (44)	41	19–35	0, 7, and 28 days	Experimental infection	CD4^+^ T cells	Prior CD4^+^ T cell immunity correlates with reduced viral shedding
Memoli et al. 2016 (45)	65	NA	0–48 days	Experimental infection	NAI	NAI baseline > 1:40 correlates with reduced severity, duration and viral shedding
Park et al. 2018 (46)	65	18–50	0 and 8 weeks	Experimental infection (pdmH1N1)	NAI and hemagglutinin (HA)-stalk antibodies	HA-stalk antibodies reduce viral shedding (duration and load) and number of symptoms but not symptom severity and duration. Baseline NAI was a stronger correlate of reduced disease severity
Gould et al. 2017 (47)	47	18–45	−1 and 29	Experimental infection (pdmH1N1)	IgA	Local sIgA not serum HAI correlates with protection from symptomatic infection
**Vaccination studies**						
McElhaney et al. 2006 (48)	100	60+	0, 4, and 10 weeks	Inactivated influenza vaccines (IIV) and natural infection	T cells	Increased T cell responses, not HAI, and IFNγ:IL-10 ratio correlated with reduced risk of infection in the elderly
Dunning et al. 2016 (49)	5,599, and 402 cases	>65	28 days	Phase III/IVb trial of IIV standard vs high dose, natural infection	NAI	HAI has limited value when viruses mismatched, NAI correlated with reduced infection cases
Clements et al. 1986 (50)	163	NA	NA	IIV, live-attenuated influenza vaccines (LAIV), experimental infection	NAI	IIV induced protective serum HAI and NAI, LAIV induced protective local HAI and NAI IgA
Jegaskanda et al. 2016 (51)	58 (IIV), 16 (LAIV), 9 (natural infection)	2–70	0, 28, and 56 days	IIV, LAIV, experimental infection	ADCC	ADCC Ab increased by IIV >1:320 reduced symptoms
Belshe et al. 2000 (52)	222	1.2–6	0–4 days	LAIV, natural and experimental infection	sIgA	LAIV was effective against natural H3N2 and FluB infection, and H1N1 challenge due to higher titers of strain-specific sIgA
Forrest et al. 2008 (20)	2,172	0.5–3	0, 7–10 days	LAIV, natural infection	T cells	>100 SFU/10^6^ PBMC protected from symptomatic infection
Ambrose et al. 2012 (53)	1,340	0.5–3	0 and 1 month for 3 years	LAIV, natural infection	sIgA	LAIV variably induces strain-specific sIgA which correlates with reduced symptomatic infection
Lillie et al. 2012 (54)	22, and 7 cases	18–45	−1, 1, 4, 7, 66, 120, and 210 days	MVA-NP + M1, experimental infection	CD8^+^ T cells	T cell activating vaccine reduced symptom severity and viral shedding
Lambkins et al. 2016 (55)	176	25 average	0, 28, 39 (post vacc.), E56, and 73 (post chall.) days	Proteasomal-IIV nasal, experimental infection	sIgA	2 dose P-IIV had 100% protection against symptomatic infection

**Table 2:** Clinical trial phase, size, scale, and influenza vaccines in development.

Phase	Preclinical	I	II	III	IV (post-market)
Purpose	Method of action	Safety and dosage	Safety and Immunogenicity	Efficacy	Post marketing surveillance
Sample size (median, range)^a^	TC, animal, human studies	72 (12–780)	217 (8–4,560)	601 (20–43,695)	170 (7–31,989)
Total no. of studies (no. and % with industry funding)^a^	–	149 (92, 62%)	230 (177, 77%)	236 (216, 92%)	184 (99, 54%)
No. vaccines in development^b^	1,000+	61	189	52	237
e.g., influenza vaccines^b^	HA-signal VLP, HA-stem, Wyeth/IL-15/5flu	LAIV H7N9	MVA NP + M1, Biondvax conserved peptide with Al(OH)_3_	IIV H5N1 with AS03	IIV H1N1 with adjuvant MF59; FluBlok

*^a^Included studies on ‘Influenza’ or ‘Influenza vaccine’ listed in ClinicalTrials.gov (56)*. *^b^Extracted from WHO Tables on clinical evaluation of influenza vaccines (57)*.

Live-attenuated influenza vaccines have been shown to induce T cell responses in children but not adults (58), and in particular children under 10 years of age have greater T cell boosting (59). In mice, early-priming preserves optimal influenza-specific CD8^+^ T cell function and diversity and protects against age-related immune decline (60), with similar age-associated effects of VE observed for LAIV (20). Furthermore, the thymus involutes during puberty greatly reducing naïve T cell output, while “inflammaging” impacts T cell priming (61). In another study, memory CD8^+^ T cells have been observed and stable longitudinally for over more than a 10-year period, most likely due to multiple re-infections since childhood (27). It is likely that repeated boosting of T cell immunity to conserved, immunodominant epitopes of NP and M1 antigens (62) results in long-term maintenance of T cell responses associated with protection from symptomatic infection (2). In older adults, individuals who received 3–4 years of annual repeated IIV vaccination, rather than single vaccination, had higher response magnitude, long-term durability, and multifunctional quality cross-reactive memory CD4^+^ T cells (63). Indeed, the T cell-based modified vaccinia virus Ankara (MVA) vector expressing NP + M1 influenza vaccine (MVA-NP + M1) could boost antigen-specific T cell memory responses in adults over 65 years of age (64). The promising MVA-NP + M1, which is currently in phase II clinical trials (Table 2), has been proposed to be used in conjunction with current IIV (65) and shown to broaden both humoral and cellular immunity. Therefore, a window for priming optimal T cell immunity with longevity exists and could be considered for vaccine design to maintain effective T cell immunity.

In humans, the lungs are enriched with CD8^+^ T resident memory (T_RM_) that rapidly generate effector cytokines upon influenza infection (66). Furthermore, prime-pull strategies have been tested in mouse studies to seed local T_RM_ responses (67), whereby a vaccine is given first parentally, i.e., intramuscularly like traditional vaccination route, to prime the T cell responses; and then inflammatory or secondary vaccine is given locally, i.e., intranasally, to pull responses to the lung, but this has had limited effect in the lung where cognate antigen presentation is required to maintain T_RM_ (68).

However, the protective efficacy of localized T_RM_ responses vs. circulating responses can only be tested in animal models. A 7-month limitation of protection has been identified by lung-resident T_RM_ in mouse models (69), but may not reflect the decay of human peripheral T cell memory in labeling and tracking studies (70). Whether lung T_RM_ have a similar critical role in modulating disease outcome in humans is unknown, but it is essential to be understood for optimal vaccine design.

### Stalling of the HA-Stalk

Development of next-generation vaccines that provide broader immune responses will be needed to protect against influenza pandemics and zoonotic influenza infections. A consensus on immune arms which are capable of providing broader immunity are split over a dichotomy, which are focused on either the anti-HA-stalk antibodies or T cell immunity. Theoretically, a HA-stalk vaccine has an exciting and promising potential, with subtype specific, multigroup, and even pan-influenza A and B antibodies being identified (71). Impressive *in vitro* and animal studies have shown the breadth of HA-stalk antibodies, yet passive transfer in human clinical trials have shown high concentrations are needed but with little therapeutic effect (72). While HA-stalk antibodies have been found to be enriched in some individuals infected with the 2009 H1N1 pandemic virus, these antibodies are notoriously low in frequency with universal antibodies such as F10 representing only 0.001% of circulating antibodies (73). There is a paucity of data on the protective role of HA-stalk antibodies in human infection studies (46) (Table 1). A human challenge study found that higher baseline level hemagglutination inhibition (HAI) antibodies were accompanied by increased HA-stalk-specific antibodies and reduced viral shedding but not symptom severity, while anti-neuraminidase (NAI) antibodies were the strongest correlate of protection (CoP) for symptomatic infection (46). Therefore, the independent role of HA-stalk antibodies remains to be defined separately from HAI and NAI antibodies. Ultimately, harnessing HA-stalk-specific B cells capable of universal immunity may also require repeated boosting to overcome immune waning, which limits the duration of current IIV.

### Alternative Strategies in Development

There are a large number of universal vaccine strategies in development in animal models, and only 61 in phase I and 189 in phase II clinical trials (Table 2) which are designed for pandemic potential viruses (56), and in total only 12.8% of these are designed to be effectively T cell stimulating (74). Apart from HA, additional viral proteins including the NP, NA, M1, and M2, are proposed to be possible targets for universal vaccines (75). Cross-reactive antibodies against these viral proteins from different subtypes have been identified and they are shown to have heterosubtypic protective effects in animals and humans. Various strategies using recombinant proteins/peptides, recombinant DNA, recombinant RNA, virus-like particles, viral vectors, and synthetic viruses for inducing heterosubtypic protective effects have been reported. Some of these approaches do not only aim at inducing broadly reactive antibodies but also cross-reactive T cell immunity against influenza infections. Clinical trials of experimental vaccines, such as proteasomal adjuvanted IIV by nasal delivery and MVA-NP + M1 have been assessed by experimental challenge and immune correlates of protection evaluated (Table 1). Previous reports also show many experimental vaccines are not undergoing clinical trials or approved for human use, suggesting a bottleneck to preclinical development (76), which could be attributed to limitations of some animal models to show vaccine efficacy or support needed from industry funding for increasing scale of clinical studies (Table 2).

## Hurdles in Extending Experimental Findings to Community: Identification of Immune Correlates of Protection

### Correlating Immune Responses to Infection and Illness Severity

Hemagglutination inhibition and single radial hemolysis assays are the only accepted serological methods used both in the US and Europe for accelerated licensure of seasonal IIV and only recognized immune CoPs for influenza currently (77–79). Other candidates of CoPs (Table 1) have been evaluated against experimental or natural human influenza virus infections and vaccine efficacy studies. The route of vaccination (intramuscular or intranasal) determines systemic vs. local immunity, and variability in sampling techniques at the mucosa may hinder the precise evaluation of mucosal antibody responses (53). Furthermore, different CoPs may be identified depending on the outcome measure that is used across the spectrum of severity, for example, from asymptomatic infection to severe illness leading to hospitalization. Therefore, the context under which each CoP was determined should be considered, and a comprehensive analytical approach is needed for clinical studies (80).

Cellular immunity is important for protection from clinical disease. For example, the Flu Watch study highlighted NP-specific CD4^+^ and CD8^+^ T cells correlated with lower nasal viral shedding (2). Other studies identified dysregulation of cytokines (namely, IL-10, MCP3, and IL-6) (35) and reduced cellular responses (including T, NK, and MAIT cells) (39, 40, 42) are associated with severe disease. The baseline presence and increasing titer of secreted IgA (33, 47) and NAI (30, 32, 45, 46) have also been identified across studies and appear as more effective correlates of protection from symptomatic infection than HAI. In addition, reports are emerging that HA-stalk (46) and antibody dependent cellular cytotoxicity (ADCC)-activating antibodies (51) have been associated with reduced viral titers upon infection. de Vries et al. showed HA-stalk-specific ADCC responses were boosted in children post-infection (81), while H7-cross-reactive ADCC antibodies were cumulative and detectable from 2 years of age but plateauing by 17 years of age (82). Early exposures to influenza boost ADCC antibodies, while older adults have limited rises in ADCC antibodies post-infection (83). A titer of HA-specific ADCC antibodies >320 correlated with reduced risk of infection, symptom scores, and viral shedding in a human challenge study (51).

While a HAI titer of 40 is believed to provide 50% protection from symptomatic infection (84), new thresholds are being defined for T cell immunity. IIV does not effectively boost T cell immunity, hence the need for new universal vaccines. The longevity derived from memory generated by natural infection is also limited, with repeated infection during our lifetime, estimated every 2–10 years (2, 27). Therefore, universal vaccines will need to do better than nature to provide longer duration immunity from symptomatic infection. From community cohort studies with baseline samples prior to symptomatic infection, Hayward et al. (2) defined the protective threshold for symptomatic infection as >20 SFU/10^6^ PBMCs (by stimulation with overlapping peptides for NP/M1); and from a LAIV children cohort study Forrest et al. (20) defined protective T cell threshold for symptomatic influenza infection as evaluated by ELISPOT was >100 SFU/10^6^ PBMCs. Inactivated vaccination from the study by Koutsakos et al. resulted in a modest boost of influenza-specific CD4^+^ T cells, while CD8^+^ T cells were not boosted (85). While CD4^+^ T follicular helper cells (Tfh), correlate with greater antibody production and HAI titers (86), and are therefore important for current IIV efficacy. Future universal vaccines need to overcome limited immunogenicity of inactivated and LAIV vaccines by more immunogenic vaccine vectors (74). While universal vaccines, such as MVA-NP + M1, which uses systemic vaccination of a one-step replication vector encoding conserved NP and M1 proteins, boosted influenza-specific CD8^+^ T cells in adults over >65 years of age (64), a notoriously difficult population for increased cellular immunity. Therefore, universal vaccines in development already show improved ability to establish T cell memory.

The immune correlates of protection from influenza are mostly derived from the comparison of infected subjects on a spectrum of severity (Table 1). However, there is a difference between correlates of protection against all infection vs. correlates of protection against symptomatic infection. Furthermore, studies of current IIV for boosting of T cell responses as correlates of protection are not ideal as IIV is not designed to stimulate cellular immunity and can impinge the cellular immunity that is being developed during natural infection (87). Rather, studies of uninfected but exposed and asymptomatic cases (low or no viral shedding) from naturally acquired infection could define immune correlates on a larger scale than possible with human challenge studies (Table 1) (2, 29, 30).

### Limitations by Prior Immunity

Prior immunity may impact vaccine efficacy, original antigenic sin, and similarly “HA-imprinting” may skew antibody and CD4^+^ T cell helper profiles by the viral subtype in the first exposure (88, 89). Furthermore, the level of neutralizing antibodies in a population will affect influenza transmission, and Bolton et al. proposed that T cell activating vaccines will have different efficacy depending on the population’s prior immunity (90). For example, due to prior immunity to seasonal H3N2 viruses but not to avian H7N9 viruses, a T cell-activating vaccine would be more efficacious for H7N9 viruses. Vaccinating an immune population with biased prior immunity may reduce vaccine efficacy, and universal vaccine strategies may differ by age group due to HA imprinting and immunosenescence. Therefore, use of a universal vaccine in younger demographics could exploit immunological imprinting to their advantage. Previously, high-antibody titers generated from childhood influenza infections which were maintained have been seen to be cross-reactive to antigenically drifted strains (91, 92).

On the other hand, seasonal influenza vaccination history may not always play a positive role in heterologous protection against subsequent influenza infection. Bodewes et al. (87) have compared the influenza A virus-specific cellular and humoral responses between 14 annually immunized children with cystic fibrosis and 27 unvaccinated healthy control children during winter season 2009–2010. A similar level of influenza-specific CD4^+^ T cell responses and neutralizing antibody titers were found between vaccinated and unvaccinated groups of children, but an age-dependent increase in the frequency of virus-specific CD8^+^ T cells were only observed in unvaccinated children. These findings indicated repeated annual influenza vaccinations might hamper the development of influenza A virus-specific CD8^+^ T cell immunity. One report in mice recently from Rowell et al. (93) also addressed such issue, which presented varied heterologous protection from a candidate universal influenza vaccine (A/NP + M2-rAd) following a history of conventional IIV vaccination. Interestingly, they found that humoral and cellular responses induced by universal vaccine could be enhanced, inhibited, or unaffected by selected prior vaccinations, and such variations may be affected by many factors including vaccine preparation and specific vaccine components.

### Standardization of Assays and Findings Across Studies

Community cohort studies to identify natural influenza virus infections and measure immunity are established in the UK (2), US (94), Vietnam (95), Hong Kong (96, 97), China (98), and Nicaragua (99) with recruitment and experiments ongoing, making this area of research an exciting area to watch. The seasonality of influenza and year-to-year variation in infectivity of viruses requires these studies to span multiple years to generate robust data, for example, the Flu Watch study spanned 2006–2011 to capture 205 infections from baseline responses (2). Peripheral blood sampling will continue to be a proxy for cellular immune correlates for influenza virus infection, and simplified and standardized assays for immune signatures or biomarkers may aid future vaccine trials (Table 2).

One of the challenges in conducting studies to identify new correlates of protection is the sample size required. Typical community-based studies can follow up more than a thousand people over multiple years (2, 30, 97), measuring immune status before the season as baseline immunity and then identifying infections after influenza activity. Dunning et al. commented that data from 1,000 to 2,000 persons may be needed for a reasonably precise estimate of an influenza CoP (49). However, such sample size is logistically challenging, and the size scale of existing studies ranged from 16 to 226 infected individuals to stratify cases by severity to derive immune correlates (Table 1), and community cohort studies such as those by Sridhar et al. (25 cases from 342 participants) (29), Hayward et al. (205 cases from 1,414 participants) (2), and Couch et al. (226 cases from 1,509 participants) (30). The scale of vaccine efficacy trials precludes many vaccine studies, especially considering the need to show an improved standard of care from current IIV, which can be reasonable when well-matched viruses are in circulation but are limited for novel viruses.

The French Interior Milieu project (100) has provided baseline immune responses of 1,000 individuals over 2 time-points, sampled the individual’s genetic background, skin biopsy, nasal swab, urine, and fecal samples, and uses 10 unique panels by flow cytometry, and 40 stimuli for characterizing adaptive and innate cellular responses. The panels measure in parallel innate cells and adaptive cells, including innate lymphoid cells, NK cells, mucosal associated invariant T cells, dendritic cells, neutrophils, B cells, and T cells (1, 2, 17, reg). Stimulation determines the individual’s ability to respond to viral, microbial, agonists, and ligands, such as influenza and Sendai viruses, *Helicobacter pylori*, Poly I:C, Flagellin, TNFα, and CD3 + CD28 (101). The implementation of standardized assays, such as the stimulation of PBMCs with influenza viruses directly at blood collection by TruCulture tubes, was essential for multicenter experimental success (102). However, due to the use of an archetypal and outdated laboratory strain, A/Puerto Rico 8/1934, the results in regard to determining relevant baseline influenza virus-specific immunity were obsolete. The study protocols from the Interior Milieu project are now being extrapolated to other ethnicities and countries to provide a spectrum of a “healthy” baseline immune system and may provide a model for assays on a larger scale beyond HAI needed for universal vaccine design. The feasibility and scale of larger community cohort studies is beyond the capacity of a single research group for processing, storage, and experimental measures (80, 103) and needs commercial partners. Therefore, consensus and synergy with other established cohorts and network design to share expertise is essential to get past the bench to define quantifiable thresholds of immune correlates of protection.

## Maximizing the Use and Effectiveness of Influenza Vaccines in the Community

Once a vaccine has been licensed to be truly effective within a population a certain coverage threshold must be reached. In 2009, the European Centre for Disease Prevention and Control set out to achieve 75% influenza vaccination coverage in the elderly and those suffering from chronic medical conditions by the winter season 2014/15. However, this target was only reached by one EU Member State in the 2013/14 season and during the 2014/15 influenza season no Member States were able to reach this coverage rate (104). Whereas the vaccination coverage rate for adults aged 18-64 years is even lower, reaching only 36.7% during 2013–2014 in the US (105). This demonstrates that current approach to vaccination, in the case of influenza, is insufficient and even with the development of novel vaccines, strategies for their implementation needs to be carefully considered.

### Considerations and Strategies to Increase Accessibility and Uptake

The live-attenuated influenza vaccine only represents 8% of the vaccine market share (106), and production in the US has been threatened by low VE in recent years. Other enhanced influenza vaccines, such as QIV Fluzone by intradermal vaccination (107), have also been threatened by a dwindling market share. Combination and heterologous approaches may complicate adherence to vaccine schedules. Various methods have been developed to stimulate HA-stalk antibodies, such as HA-headless or chimeric HA, and combination strategies of prime boost for four doses (108). However, anti-HA-stalk antibody-stimulating vaccine regimes by heterologous prime boost used in mouse studies to elicit HA-stalk antibody responses may not be feasible in practice in the community, with each regime requiring separate licensure and multiple doses reducing vaccine adherence. The human papilloma virus (HPV) and hepatitis B virus (HBV) vaccines both require a homologous 3-dose regime within 2 years for optimal sero-protection, and HBV also requires a 10-year dose booster. Adherence to HPV vaccine 3-dose schedule is only 28% (109), and similarly 29% for HBV vaccination (110). A comprehensive vaccination record system will be instrumental for orderly vaccination schedules.

An increasingly difficult barrier to successful vaccination strategies is “vaccine hesitancy.” The WHO Strategic Advisory Group of Experts (SAGE) on Immunization has defined vaccine hesitancy as a “delay in acceptance or refusal of vaccination despite availability of vaccination services” (111). Vaccine hesitancy can develop into refusal and the encouragement of others to refuse vaccination, leading to unvaccinated clusters within a community and severe public health consequences. One of the more concerning effects of vaccine hesitancy is the effect on vaccination coverage in HCWs. Vaccination for HCWs is recommended in most countries but mandatory vaccination programs vary. A survey of HCWs in China found a coverage rate of only 9.5% in the seasons of 2009/2010 to 2011/2012 (112). However, vaccination in HCWs in the US has increased since the 2010/2011 season, reaching 64.8% in 2014/2015 (113), demonstrating this issue varies greatly by country due to policy decisions and cultural factors. If HCWs themselves are hesitant about current vaccines, novel vaccines that utilize “non-traditional” approaches for universal immunity may require extensive explanation and promotion to HCWs to encourage self-vaccination and increase vaccine recommendations to patients.

### Indirect Protection in the Community With Vaccine Uptake

Many studies have shown that increasing vaccination uptake in children and younger adults reduces influenza burden in older adults (114–116, 117). Older children and adolescents have been shown to be the key age groups affecting the initial spread of influenza infections within a community (118). Elderly individuals often come into contact with children and young adults in household and urban settings, public areas and transportation. One of the clearest examples of this was seen in Japan, when influenza vaccination of school children ceased in 1994, leading to an increase in elderly mortality rates (119). A study analyzing US vaccine data also found that in areas where there was ≥31% vaccine coverage in younger adults, the elderly had a 20.6% lower chance of being diagnosed with influenza than in areas with a ≤15% coverage rate (120). Vaccination of healthy children could also form the basis of establishing early T cell memory and broader HA imprinting from an immunological perspective.

## Conclusion

**Figure 1:**
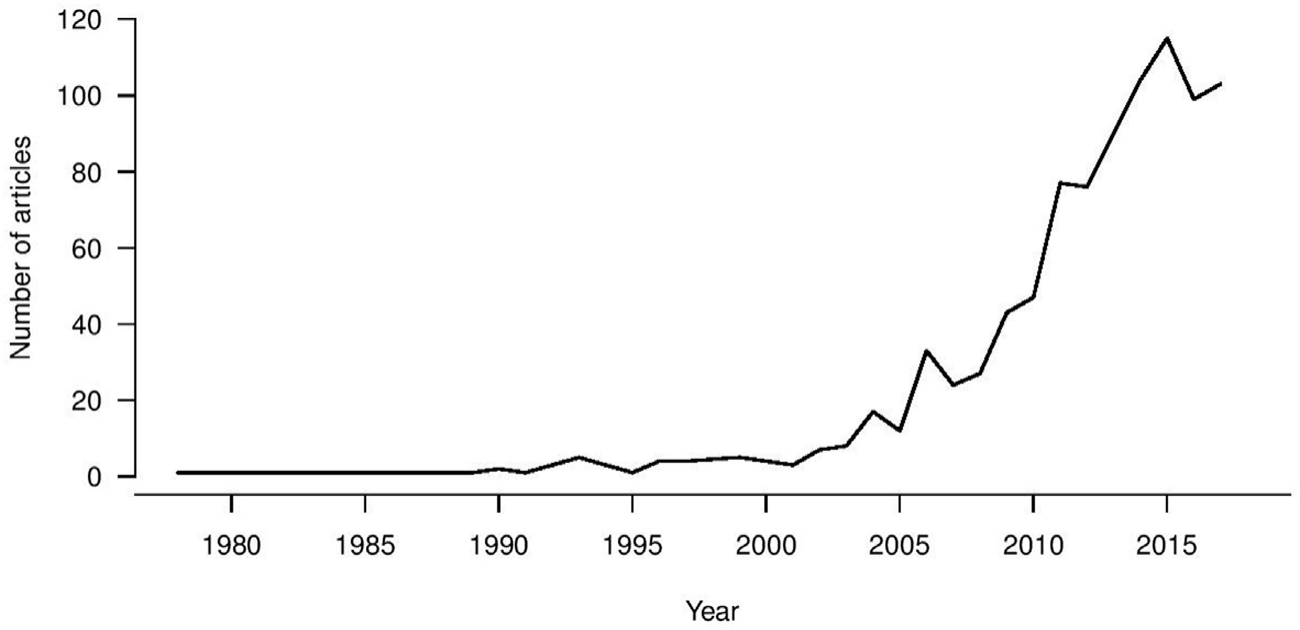
Pubmed indexed publication trend for universal influenza vaccines.

Universal influenza vaccine research is a growing trend (Figure 1), with first reports in the 1970s of heterologous immunity in the absence of antibodies for recombinant vaccines being developed following the antigenic switch from H1N1 to H3N2 viruses (121). A large increase in the universal vaccine research field has been seen since 2003, coinciding with zoonotic infections from avian and equine sources and pandemic viruses becoming a real threat to public health. Therefore, the drive for increased breadth of coverage for influenza vaccine has been a long-term objective, and the recent NIAID push has been a “call to arms” to address this issue. An increasing number of immune biomarkers that are associated with protection against influenza virus infection and disease severity *in vitro* and *in vivo* have been identified, leading to vaccines designed to elicit these immune markers at different stages of clinical trials. Such a strategy assumes that these markers are correlates of protection in humans, but whether such assumptions hold is yet to be confirmed in large epidemiological studies. Ultimately, immune correlates should be compared in parallel and defined within a weighted hierarchy to drive vaccine design which can stimulate multiple immune arms effectively. Alternatively, despite measurable influenza-specific T and B cell immunity, all healthy adults experience repeat infections in their lifetime. Additionally the WHO goals to promote longevity of responses may also require a vaccine that elicits a “better than nature” response. With increased attention and funding for this area, particularly from the National Institutes of Health, there is real hope for the successful development of universal influenza vaccines.

## Author Contributions

SV, NL, MB, YLM, AL, LP and BC wrote and prepared the review. SV and NL prepared the figures and tables.

## Conflict of Interest Statement

BC has received research funding from Sanofi Pasteur, and honoraria from Sanofi Pasteur and Roche. The authors report no other potential conflicts of interest.
